# Stem Cell and Macrophage Roles in Skeletal Muscle Regenerative Medicine

**DOI:** 10.3390/ijms221910867

**Published:** 2021-10-08

**Authors:** Pasqualina Scala, Laura Rehak, Valentina Giudice, Elena Ciaglia, Annibale Alessandro Puca, Carmine Selleri, Giovanna Della Porta, Nicola Maffulli

**Affiliations:** 1 Department of Medicine, Surgery and Dentistry, University of Salerno, Via S. Allende, 84081 Baronissi, Italy; pscala@unisa.it (P.S.); vgiudice@unisa.it (V.G.); eciaglia@unisa.it (E.C.); apuca@unisa.it (A.A.P.); cselleri@unisa.it (C.S.); nmaffulli@unisa.it (N.M.); 2 Athena Biomedical innovations, Viale Europa 139, 50126 Florence, Italy; laurarehak@gmail.com; 3 Hematology and Transplant Center, University Hospital “San Giovanni di Dio e Ruggi D’Aragona”, Largo Città d’Ippocrate 1, 84131 Salerno, Italy; 4 Clinical Pharmacology, University Hospital “San Giovanni di Dio e Ruggi D’Aragona”, Largo Città d’Ippocrate 1, 84131 Salerno, Italy; 5 Cardiovascular Research Unit, IRCCS MultiMedica, Via Milanese 300, 20138 Milan, Italy; 6 Interdepartment Centre BIONAM, University of Salerno, Via Giovanni Paolo I, 84084 Fisciano, Italy; 7 Centre for Sports and Exercise Medicine, Barts and The London School of Medicine and Dentistry, Queen Mary University of London, 275 Bancroft Road, London E1 4DG, UK

**Keywords:** skeletal muscles, trauma, muscle healing and repair, stem cells, macrophages, immune cell role in tissue repair

## Abstract

In severe muscle injury, skeletal muscle tissue structure and functionality can be repaired through the involvement of several cell types, such as muscle stem cells, and innate immune responses. However, the exact mechanisms behind muscle tissue regeneration, homeostasis, and plasticity are still under investigation, and the discovery of pathways and cell types involved in muscle repair can open the way for novel therapeutic approaches, such as cell-based therapies involving stem cells and peripheral blood mononucleate cells. Indeed, peripheral cell infusions are a new therapy for muscle healing, likely because autologous peripheral blood infusion at the site of injury might enhance innate immune responses, especially those driven by macrophages. In this review, we summarize current knowledge on functions of stem cells and macrophages in skeletal muscle repairs and their roles as components of a promising cell-based therapies for muscle repair and regeneration.

## 1. Introduction

**Figure 1:**
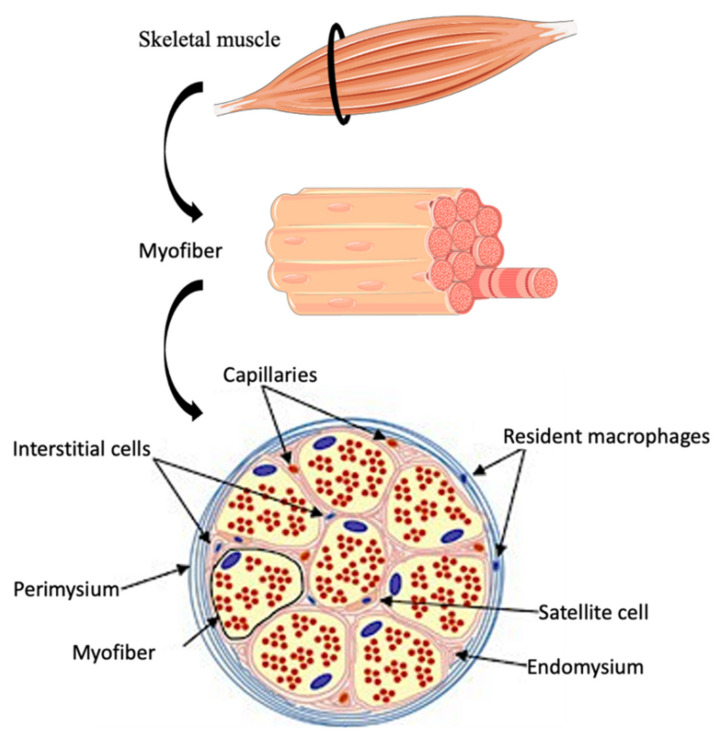
Schematic representation of the skeletal muscle structure. The connective tissue, called perimysium, surrounds groups of fibers, organized in multinucleated and longitudinally aligned bundles. Each single muscle cell, or myofiber, is surrounded by endomysium. Satellite cells are located between the connective tissue sheet and the myofiber plasma membrane, called sarcolemma.

Skeletal muscle regeneration (SkMR) is the ability of injured muscles to functionally recover after traumas and is related to the intrinsic healing properties of injured tissue and to the type of injury based on the number of involved myofibers, muscle strength, and loss of contractility [1,2,3]. SkMR is a complex and finely regulated biological process involving different cellular populations, such as inflammatory cells and muscle stem cells, also known as satellite cells due to their spatial localization between connective tissue layers and sarcolemma (see also Figure 1) [4]. Satellite cells are quiescent in steady-state conditions; however, after injuries, they proliferate and differentiate to restore skeletal muscle physiology by sequential expression of specific transcription factors, such as Paired box 7 (*Pax7*) [5,6,7,8], followed by myogenic regulatory factors (MRFs), Myoblast determination protein (*MyoD*), Myogenic factor 5 (*Myf5*), Myogenic factor 6 (*Myf6*), and finally Myogenin (*Myog*). *MyoD* and *Myf5* are overexpressed during myoblast proliferation. While *MyoD* downregulation is replaced by *Myf6* and *Myog*, triggering terminal differentiation of muscle progenitors towards elongated myocytes that fuse in multinucleated myotubes and mature in myofibers [9,10]. Fibro-adipogenic progenitors (FAPs) are important in SkMR and can negatively or positively influence muscle recovery depending on microenvironment composition [11,12]. For example, interleukin (IL)-1β inhibits FAP differentiation, while IL-4 has a pro-adipogenic effect; conversely, IL-15 stimulates FAP proliferation and prevents adipogenic differentiation [11,13]. Once activated, FAPs phagocyte necrotic debris, favor revascularization, release extracellular matrix (ECM) components, and promote matrix remodeling [14,15]. In chronic injuries, muscle tissue can be replaced with a mix of white adipocytes and fibrotic cells in a process called fatty degeneration, in which satellite cells can differentiate in both fibrocytes and adipocytes. When physiologic myogenic differentiation is impaired due to cell defects or pathological environmental changes, satellite cells switch to an alternative differentiation pathway [12,16,17,18]. In several in vitro and in vivo studies, successful muscle healing has been described when both stem cells or inflammatory cells are activated and participate in the regeneration processes [4]. The present review provides an update of stem cell and macrophage involvement in SkMR.

## 2. SkMR Biology

**Figure 2:**
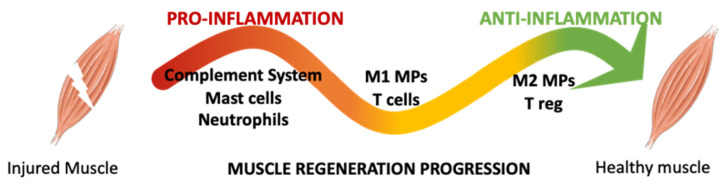
Schematic representation of the events succession related to immune cells during the SkMR. In early phase, the innate immune response activates the complement system, mast cells, and neutrophils. All these cells recruit monocytes at the injured site that mature in macrophages (MPs) with first a pro-inflammatory phenotype (M1-MPs) and then an anti-inflammatory phenotype (M2-MPs). These cells cooperate with local stem cells to promote tissue repair and regeneration.

The SkMR process can be schematically divided into three phases: (i) destruction, (ii) repair, and (iii) remodeling. In the first phase, the injured necrotic site is spatially defined within the contraction band composed of condensed cytoskeletal material and necrotic cells that release intracellular components from sarcolemma disruption into the bloodstream [19,20]. Injury also compromises blood vessel integrity leading to the activation of coagulation and complement cascades and causing muscle-resident mast cell-dependent inflammatory responses [21]. In the second phase, necrotic tissue is cleared by phagocytosis; then, during the third phase, myofiber regeneration begins and proceeds to the full recovery of skeletal muscle contractility and structure [19]. In all those phases, the immune system is strongly involved, influencing the rate of the healing process and scar tissue formation (Figure 2). After a few seconds post-injury, the complement system is rapidly activated by several damage-associated molecular patterns, such as the heat shock protein and high mobility group box-1 protein (HMGB1), released by injured myofibers [22,23]. Complement cascade activation results in the recruitment of immune cells at the site of injury and the start of inflammatory responses. Indeed, it has been reported that a deficiency of complement proteins, especially C3a, results in an impaired regeneration with smaller myofiber formation [22]. Leukocyte recruitment is directed by C5a at the injured site and by mast cells that rapidly release several pro-inflammatory mediators, such as tumor necrosis factor (TNF)-α, histamine, IL-1, IL-6, platelet-activated-factor, and prostaglandins [24,25]. Resident neutrophils are also quickly activated after injury and release pro-inflammatory cytokines, including TNF-α, IL-1β, and interferon-γ (IFN-γ). Within a few hours after the event, other neutrophils are recruited by secreted factors and contribute to oxidative and proteolytic modifications in the injured area [26,27]. Neutrophil depletion significantly decreases macrophage accumulation at the injured site, suggesting their fundamental role in monocyte recruitment [28]. 

In severe injuries, such as muscle tears, endogenous muscle repair capacity is not sufficient for complete muscle recovery, and clinical management of these conditions remains one of the most challenging in the orthopedic field. In these cases, therapeutic approaches include cell-based therapies using stem cells of myogenic and non-myogenic origins (see Table 1) [29]. 

In more detail, stem cell therapy (SCT) can employ satellite cells because they can repopulate the stem cell niche increasing the regenerative muscle potential and its contractility [30,31,32,33,34]. Moreover, muscle-derived stem cells (MDSCs), not-terminally differentiated satellite cell precursors, have mesodermal tissue commitment potential and elevate engraftment rates after transplantation [35,36]. However, the efficacy of SCT in SkMR is still limited because satellite cells and MDSCs are rare populations (2–7%), and their isolation and harvesting are extremely challenging [29]. For these reasons, mesenchymal stem cells (MSCs) are often preferred, even though they are not of myogenic origin. MSCs have a broad differentiation potential including bone [37], cartilage [38], tendon [39,40,41], and muscle [42], and can be mainly isolated from several tissues, such as bone marrow (BM) and adipose tissue [43]. Recently, also MSCs from the umbilical cord seemed an interesting cell source for musculoskeletal tissue engineering [40,41].

In particular, BM-derived MSC (BM-MSC) therapy can significantly improve muscle contraction strength, as well as adipose tissue-derived MSC (ADSC) infusion, which can increase the number of new regenerated myofibers at the injury site [44,45,46,47]. Moreover, BM-MSCs can downregulate pro-inflammatory cytokines while upregulating anti-inflammatory mediators reducing fibrosis evolution through transforming growth factor-β (TGF-β) signaling and related collagen deposition [48]. The failure of BM-MSC engraftment is associated with massive and prolonged leukocyte infiltration in the muscle, reduced myofiber regeneration, increased cell necrosis, and elevated pro-inflammatory cytokine secretion [49].

## 3. Macrophages and Muscle Healing 

**Figure 3:**
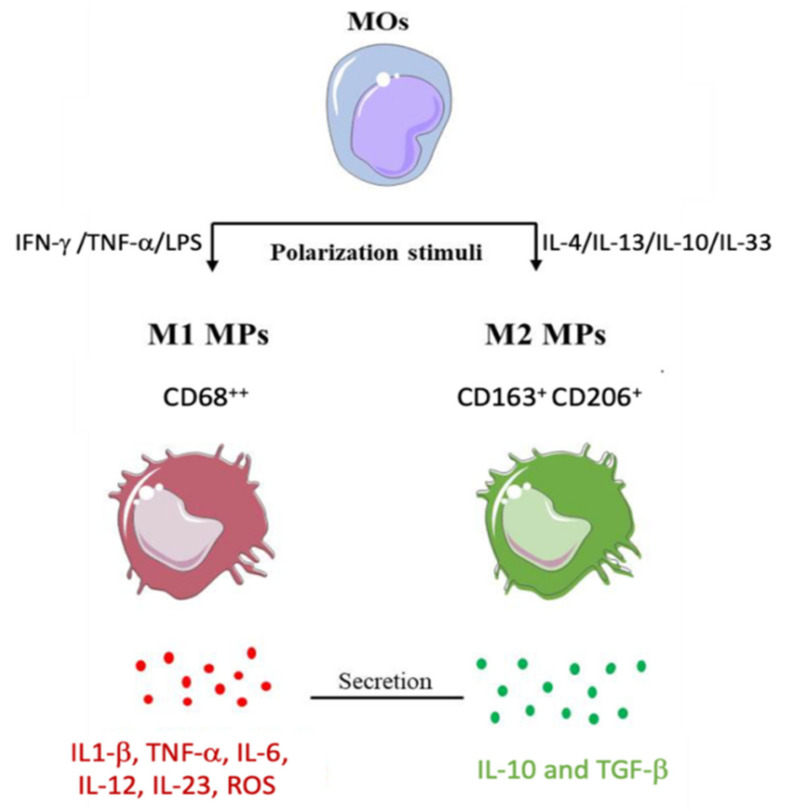
Schematic representation of macrophages polarization protocols adopted in vitro. Monocytes (MOs) evolve towards the pro-inflammatory (M1) macrophages (MPs) after T-helper 1 (Th1) response cytokines or microbial stimuli, such as lipopolysaccharide (LPS); conversely, they evolve towards anti-inflammatory (M2) macrophages (MPs) after T-helper 2 (Th2) cytokines. M1-MPs are characterized by a high expression of CD68 surface markers, the secretion of pro-inflammatory cytokines and reactive oxygen species (ROS). M2-MPs are characterized by elevated levels of CD163 and CD206 and low levels of CD68; they also secrete anti-inflammatory cytokines. Abbreviations: IFN-γ, Interferon- γ, TNF-α, Tumor Necrosis Factor- α, IL, Interleukin, TGF-β, Transforming growth factor-β.

Macrophages can polarize toward two different phenotypes based on acting stimuli (Figure 3): pro-inflammatory (M1-MPs) and anti-inflammatory (M2-MPs) cells. T helper (Th)1-related cytokines, such as IFN-γ and TNF-α, or lipopolysaccharide (LPS), induce M1-MP differentiation, while Th2 cytokines (e.g., IL-4 and IL-13) or IL-10 and IL-33 switch macrophage differentiation toward the M2-MP phenotype [50,51]. M1-MPs express CD68 at a high level that mediates the activation of phagocytosis and pro-inflammatory cytokine secretion; conversely, M2-MPs, positive for CD163 and CD206 and negative for CD68, promote anti-inflammatory cytokine release [51]. 

The in vivo M1/M2 dichotomy is more a dynamic process rather than an on/off differentiation as described in vitro experiments. Indeed, in vivo, macrophages can easily switch from one functional phenotype to another in response to several local signals: M1-MPs accumulate at the injured area within the 24 h, rapidly decreased, and switched to M2-MPs within two/four days [52,53,54]. Simultaneously, tissue-resident macrophages recruit neutrophils through chemoattractant proteins, such as monocyte chemoattractant protein 1 (MCP-1) [55]. Satellite cells also contribute to monocyte recruitment at the injury site via macrophage-derived chemokine (MDC) in the earliest phases of MCP-1 during the late stages of myogenic differentiation [56]. At early regeneration stages, neutrophils amplify M1-MP-mediated phagocytosis through oxidative modification of low-density lipoproteins that bind and activate CD68 [57]. After clearance of debris at the injured site by macrophages, M1-MPs secrete TGF-β, responsible for phagocytosis rate reduction [58]. The urokinase (uPA)-mediated plasminogen activation system is involved in various biological processes, including inflammation, wound healing, and muscle regeneration [59]. During regeneration, uPA-expressing macrophages promote effective muscle regeneration through ECM regulation and remodeling, as well as favoring monocyte migration at the injured site [59,60,61].

## 4. Macrophages and Muscle Healing: In Vivo Evidence

The pivotal role of macrophages during SkMR has been largely confirmed in vivo mouse models (Table 2). Chemokine C-X3-C motif receptor (CX3CR)^lo^/Ly-6C^+^ monocytes/macrophages are the first cell population invading the site within 90 min after injury reaching a peak at 24 h. They produce a high amount of IL-1β and TNF-α during the first two-three days then switching to CX3CR^hi^/Ly-6C^−^ cells expressing IL-10 and TGF-β1, characterizing the regenerative phase [58]. CX3CR^lo^/Ly-6C^+^ monocytes/macrophages mediate phagocytosis of necrotic myofibers in the first two days after injury, while Ly-6C^−^ macrophages surround the new regenerating myofibers between four-eight days after the event [62,63]. An earlier start of anti-inflammatory responses is associated with inefficient regeneration, as described in mitogen-activated protein kinase phosphatase-1 (MKP-1), a regulator of MAPK activation, deficient mouse model. In MKP-1^−/−^ mice, macrophages are still able to accumulate at the injury site; however, inflammation persists after 10 days post-injury with a constant expression of myogenic markers in satellite cells, and myofibers are of smaller size and centrally nucleated. At the injured site, both Ly-6C^+^ and Ly-6C^−^ macrophages express high levels of anti-inflammatory cytokines on day three, suggesting a premature activation of anti-inflammatory responses [64]. Protein kinase AMP-activated catalytic subunit α-1 (AMPKα-1) plays an important role in macrophage phenotype transition. In AMPKα-1^−/−^ mice, M1-MPs remain constantly increased, while M2-MP frequency does not change during muscle regeneration, resulting in impaired SkMR [65].

The role of macrophages in SkMR has been also investigated by blocking cell recruitment at the injury site and by showing the kinetics of cell recruitment on correct muscle regeneration. Macrophages are important in the first and early phase of inflammation because a deficient accumulation at the injured site results in smaller regenerating myofibers and in fatty tissue accumulation [66]. High frequency of M1-MPs at the injured site is favored by monocytes; however, monocyte depletion does not abolish macrophage accumulation likely due to intrinsic M1-MP proliferative capacity [67]. In this case, necrotic myofibers are not efficiently cleared and persist after nine days post-injury with fatty degeneration [68]. C-C chemokine receptor type 2 (CCR2), the natural ligand of MCP-1, is not expressed in healthy muscle while is released early during regeneration and is essential for macrophage recruitment [69]. In *CCR2^−/−^* mice, mononuclear cell infiltration (represented mostly by neutrophils) is minimal in early phases and necrotic myofibers are still present after twenty days with a consistent accumulation of adipocytes [70]. Moreover, lower macrophage levels in the injured area are associated with reduced insulin-like growth factor 1 and impaired muscle regeneration [71]. Similarly, *MCP1^−/−^* mice show a markedly reduced inflammation and macrophage accumulation at day three post-injury, with the presence of necrotic myofibers at day seven and small-sized regenerating myofibers detectable only at day twenty [72,73]. SkMR can be favored by macrophage injections, especially using in vitro polarized M1-MPs, within 24 h post injury resulting in significant improvement of muscle function with larger myofibers. The efficacy of macrophage injection is related to a faster clearance of necrotic debris that allows a rapid replacement of newly myofibers with faster transition from M1 to M2 [74].

## 5. Macrophages and Myogenic Precursors: A Functional Crosstalk 

**Figure 4:**
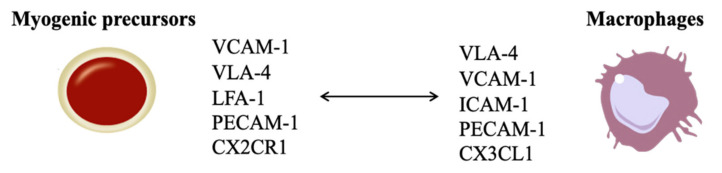
Schematic representation of myogenic precursors and macrophages crosstalk. A functional crosstalk is established between myogenic precursors and macrophages to promote cells survival and proliferation during skeletal muscle healing. Macrophages expressed the vascular cell adhesion molecule 1 (VCAM-1), intercellular cell adhesion molecule binding 1 (ICAM-1), platelet-endothelial cell adhesion molecule homophilic (PECAM-1) and C-X3-C motif chemokine ligand 1 (CX3CL1); all these signals interact with their counter-ligands on myogenic precursors: very late antigen 4 (VLA-4), leukocyte function-associated molecule 1 (LFA-1), platelet-endothelial cell adhesion molecule homophilic (PECAM-1), and C-X2-C motif receptor 1 (CX2CR1), respectively. Moreover, myogenic precursors express VCMA-1 that interact with VLA-4 of immune cells.

Biological mechanisms underlying the complex crosstalk between myogenic precursors and macrophages at the injured site remain unclear [56]; a summary of possible functional crosstalks is summarized in Table 3. Macrophages provide survival and mitogenic stimuli directed to myogenic precursor growth. During SkMR, a specific injury-located MP group creates a transient niche for satellite cell proliferation by releasing mitogenic molecules, such as cytokine nicotinamide phosphoribosyltransferase whose receptors are on satellite cells (C-C motif chemokine receptor type 5, CCR5) [75]. The high satellite cell proliferating rate is due to activation of anti-apoptotic signaling pathways, such as reduced activation of pro-apoptotic caspase-3 and increased anti-apoptotic Bcl-2 protein expression [56,76]. In detail, four cell-cell anti-apoptotic pathways are constitutively expressed by myogenic precursors and their ligands by macrophages: (i) vascular cell adhesion molecule 1 (VCAM-1)/very late antigen 4 (VLA-4); (ii) intercellular cell adhesion molecule 1 (ICAM-1)/leukocyte function-associated molecule 1 (LFA-1); (iii) platelet-endothelial cell adhesion molecule homophilic 1 (PECAM-1); (iv) C-X3-C motif chemokine ligand 1 (CX3CL1) binding to C-X2-C motif receptor 1 (CX2CR1) [76]. VCAM-1 is considered a specific marker for myogenic precursors. During myogenesis, VCAM-1/VLA-4 interaction occurs with the expression of VCAM-1 by myogenic progenitors and of VLA-4 by immune cells [77], as summarized in Figure 4.

M1-MPs inhibit myogenic precursors fusion, while M2-MPs stimulate myotube formation even without direct cell contact [78]. Moreover, the stage of the muscle healing process influences the effects of macrophages on myogenic precursors. Macrophages expressing pro-inflammatory markers are abundant in regenerating areas negative for *Myog* (a transcription factor expressed only in differentiated myogenic cells) suggesting different associations based on proliferation or differentiation of myogenic precursors [78,79].

## 6. Cytokines and Muscle Healing 

**Figure 5:**
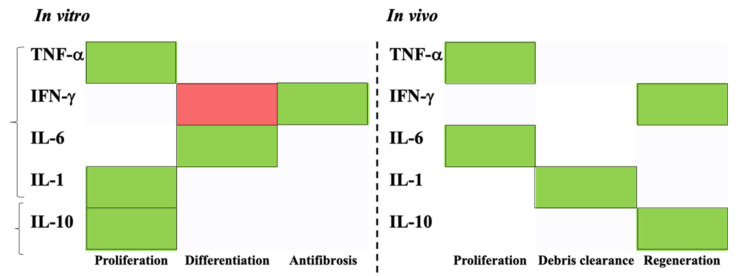
Schematic representation of cytokines contribution documented in both in vitro and in vivo studies (green box: promotion; red box: inhibition). Pro-inflammatory and anti-inflammatory cytokines showed an important contribution during skeletal muscle regeneration: in vitro, they mainly activated myoblasts proliferation and differentiation (except for INF-γ); in vivo, cytokines expression, promoted tissue clearance and its regeneration. Abbreviations: TNF-α, Tumor Necrosis factor-α, IFN-γ, Interferon-γ, IL, Interleukin.

Cytokines are also involved in the complex crosstalk between myogenic precursors and macrophages, as described below and summarized in Table 4 and Table 5, and Figure 5).

### 6.1. TNF-α

TNF-α is transiently upregulated in myoblasts within 3 to 48 h post differentiation induction in a dose-dependent manner: myogenesis is stimulated at low TNF-α concentrations, while is inhibited at high concentrations [80,81]. TNF-α has mitogenic and chemotactic effects on proliferating primary rat myoblasts [82,83]. Proliferating myoblasts fuse each other’s within 4 days in absence of TNF-α, whereas TNF-α treatments completely inhibit myotube formation and reduce *Myog* expression. In healthy muscles, TNF-α expression is constitutively low; however, after injury, its expression increases within 5 h, reaching a peak at 24 h, and then gradually decreases. In TNF-α receptor double-knockout mice, p38 MAPK expression diminishes together with *MyoD-1*, a proliferation marker, in TNF-α deficient mice [84]. Moreover, this proliferating effect is exerted on satellite cells after in vivo TNF-α intraperitoneal injection [82], while *Myog* is reduced confirming differentiation inhibition of this cytokine on myoblasts [85]. TNF-α could be also involved in muscle strength recovery, likely through modulation of muscle regulatory gene expression, such as *MyoD* [80,84].

### 6.2. IFN-γ

IFN-γ, a pro-inflammatory cytokine, favors myoblast proliferation, prevents fibrotic events in SkMR, and is expressed by proliferating myoblasts while not by differentiated cells. IFN-γ stimulation impairs myoblast fusion and differentiation gene expression, likely through inhibition of *Myog* expression by Class II Major Histocompatibility Complex transactivator (CIITA). However, this inhibition is reversible as CIITA is quickly downregulated, and muscle-specific genes upregulated [86,87]. IFN-γ also acts as an antifibrotic agent by reducing TGFβ-1 expression [88]. IFN-γ expression is at basal levels in healthy muscles, while increases after injury, peaking at day five post-injury corresponding to immune cell and myoblast infiltration. Moreover, IFN-γ is important in macrophage recruitment, induction of regenerating myofibers, and connective tissue formation [87,88].

### 6.3. IL-6

IL-6 is an important mediator in SkMR and is highly produced by myogenic cells and macrophages. IL-6 is necessary for stimulation of myoblast proliferation, and its levels progressively decrease with clearance of necrotic cells [89,90]. Myoblast proliferation is favored by low and medium IL-6 concentrations, while high concentrations induce myogenic differentiation. In addition, IL-6 shows time-dependent effects on primary cultures of human myoblasts: *MyoD* expression increases after 24 h, with subsequent increase of *Myog* at 48 h [91]. IL-6 also exerts a chemoattractant role for macrophage recruitment at the injured site [90]. In healthy muscles, IL-6 is not expressed, while increases at one day post-injury, and starts to decrease after five days from the event. In IL-6^−/−^ mice, the regenerative rate is lower because proteins related to myogenesis are poorly expressed and newly formed myofibers are smaller with interstitial fibrosis, and also because satellite cells and myoblasts show a lower proliferation and migration rate [89,90]. 

### 6.4. IL-1

IL-1 is a pro-inflammatory cytokine involved in muscle growth and regeneration probably enhancing clearance of necrotic fibers. In myoblasts, IL-1β, an IL-1 isoform, induces cyclin A and B1, master regulators of G1/S and G2/M transition, respectively. Between three to five days post-differentiation induction, IL-1β enhances muscle proteins synthesis, such as myosin heavy chain, and increases fusion index [92]. Prolonged IL-1 exposure induces muscle catabolism in a time-dependent manner with reduction of myotube width and sarcomeric actin levels [93]. Myoblasts from IL-1 knockout mice show a significantly slower growth compared to wild type. The proliferation rate can be restored with exogenous IL-1β, but not with IL-1α [94]. Moreover, inflammatory cells are fewer, necrotic myofibers are not efficiently cleared, and myogenic differentiation marker expression is markedly reduced in IL-1 deficient mice compared to controls [94]. IL-1β expression reaches a peak at two-three days after injury and remains high up to 5 days post-event [95].

### 6.5. IL-10

IL-10 is the main anti-inflammatory cytokine in SkMR and is essential for regeneration of new myofibers. IL-10 treatment does not affect myoblast proliferation, while activated macrophages and induce proliferation and differentiation of myoblasts, without affecting *MyoD* and *Myog* gene expression along the early differentiation stage [54]. IL-10 expression is upregulated three days post-injury reaching the maximum after seven days [96]. In IL10^−/−^ mice, injured myofibers are not efficiently cleared resulting in reduced centronucleated myofibers that also show smaller sizes compared to control. Moreover, in IL10^−/−^ mice, M1/M2 transition is delayed, resulting in amplification of Th1 responses and increased Myog levels, likely due to indirect effects of other cytokines [54].

**Table 4:** Cytokines and skeletal muscle regeneration. In vitro studies.

Cell Culture	Results	Ref
C2C12	After differentiation induction, TNF-α expression increases	[80]
Murine myoblasts	Myoblast migration stimulation	[82]
Murine myoblasts	Myoblast migration induction	[83]
C2C12	Inhibition of myoblast differentiation into myotubes	[81]
C2C12, Primary myoblasts	Inhibition of myoblast differentiation	[86]
C2C12	Reduction of myoblast proliferation	[87]
Muscle-derived fibroblasts C2C12	Decrease TGFβ-1 expression	[88]
Mice MPs, C2C12	Induction of myoblast proliferation	[90]
C2C12, Primary human myoblasts	Proliferation and differentiation due to different IL-6 concentrations	[91]
C2C12	Increase of myoblast fusion index	[92]
C2C12	IL-1 induces muscle catabolic pathway	[93]
Mice satellite cells	IL-1 induces cell proliferation	[94]
Mice MPs, C2C12	IL-10 activated macrophages promote myoblasts proliferation	[54]

TNF-α, Tumor Necrosis factor-α, TGF-β1, Transforming growth factor-β1, IL, Interleukin, MPs, macrophages.

**Table 5:** Cytokines and skeletal muscle regeneration. In vivo studies.

Animals	Injury	Injection	Muscle	Results	Ref
Mice	Cooled probe	-	Tibialis anterior	TNF-α involved in muscle strength recovery	[84]
Mice	-	TNF-α	Soleus Diaphragm	TNF-α stimulates satellite cell proliferation	[82]
Mice	HS/RL	TNF-α	Soleus Gastrocnemius	Decrease of Myog expression	[85]
Mice	Cardiotoxin	-	Soleus	SkMR impairment	[80]
Mice	Cardiotoxin	IFN-γR blocking antibody	Extensor digitorum longus Tibialis anterior	Reduction of regenerating myofiber formation	[87]
Mice	Laceration	IFN-γ	Gastrocnemius	Minor fibrosis rate	[88]
Mice	Cardiotoxin	IL-6	Tibialis anterior Gastrocnemius	Inhibition of proliferating cells	[90]
Mice	Overloading	-	Soleus Plantaris muscles	Stimulation of migration and proliferation	[89]
Mice	BaCl_2_ injection	-	Tibialis anterior	Early increase of IL-1β expression	[95]
Mice	Cardiotoxin	-	Tibialis anterior	Reduction of inflammatory cells infiltration	[94]
Mice	Contusion	-	Gastrocnemius	IL-10 peak at 7 days	[96]
Mice	HU/RL	-	Soleus	SkMR impairment	[54]
Mice	FAE	-	Hindlimb muscles	Necrotic myofibers persistence; fat accumulation	[73]
Mice	TK-I/R	-	Gastrocnemius	Recovery of muscle functionality by M1-MPs delivery	[74]

TNF-α, Tumor Necrosis factor-α, Myog, Myogenin, SkMR, Skeletal muscle regeneration, IL, Interleukin, HS/RL, hind limb suspension/reloading, IFN-γR, interferon-γ (IFN-γ) receptor, BaCl2, barium chloride, HU/RL, hind limb unloading/reloading, FAE, femoral artery excision, TK-I/R, tourniquet-induced ischemia/reperfusion injury, MPs, macrophages.

## 7. Conclusions and Perspectives 

When skeletal muscle regeneration remains unresolved, cell therapy could represent a valid clinical approach. Myogenic stem cells provide excellent results when infused at optimal concentrations; however, myogenic stem cells are rare and their isolation is still challenging [29]. For these reasons, stem cell therapy has moved towards other types of (mesenchymal) stem cells, harvested from various adult human tissues, such as bone marrow and adipose tissue. Similar to myogenic stem cells, also mesenchymal stem cells are difficult to obtain in optimal amounts for transplant success [97,98]. Stem cell sampling, harvesting, and preparation is even more difficult in patients with pathologies [99] worsening autologous transplantation outcomes. Stem cells are active in muscle repair because of their immunomodulatory effects, many of these still undiscovered, and because of immune system recruitment through cellular and soluble factor release. 

On the other hand, macrophages seem to be the principal immune cell involved in muscle regeneration by first favoring inflammation and clearance of injured area from necrotic debris; and then by enhancing inflammation resolution and forcing myogenic precursor cells to differentiate in regenerating myofibers. However, the complex crosstalk between macrophages and myogenic cells is still under investigation and it is still unclear if cell-cell contacts or paracrine signals induced by soluble factors are fundamental in restoring skeletal muscle physiology. Indeed, released cytokines exert a fine regulation of the muscle healing process, as pro-inflammatory molecules enhance myogenic precursor proliferation whereas anti-inflammatory ones influence macrophage transition towards an anti-inflammatory phenotype, damping inflammation. Several in vivo studies suggest that the presence of M1-MPs can accelerate clearance of necrotic debris and promote the resolution of inflammation when switched in M2-MPs. In vitro and in vivo studies -with some differences related to microenvironment composition- strongly suggest that macrophages are the main actors of muscle regeneration and that the lack of this cell subset severely impairs all steps of muscle healing. 

Available literature indicates the predominant role of the immune system in muscle regeneration that requires further and deeper investigations also because of the therapeutic potential of targeting or modulating immune cells for facilitating muscle repair. For example, peripheral blood mononuclear cells are already described as a valid alternative source for cell therapy, as they are easier to sampling and isolate [49,90]. Moreover, peripheral blood cell therapy can dramatically increase the number of regenerating myofibers at seven days after autologous transplantation by using a simple whole-blood gravity filtration device such as the device largely used in patients with critical limb ischemia and ineligible for surgical revascularization; in those cases, the treatment allowed a significant reduction in amputation rate [100,101,102]. This clinical observation is relevant to supports the role of the immune system in tissue regeneration and healing; indeed, even if further studies are required to understand the complex cellular cross-talk involved in these processes, it may open encouraging perspectives for clinical use of peripheral blood cells in skeletal muscle regeneration. 

## Figures and Tables

**Table 1:** Potential SC therapies for skeletal muscle regeneration. In vivo studies.

Animals	Transplanted SCs	Injury	Muscle	Results	Ref
Mdx mice	MuSCs	Notexin injection	Tibialis anterior	Self-renewal of host SC niche	[29]
Mice	MuSCs	Notexin injection	Tibialis anterior	High engraftment percentage	[30]
Mdx mice	MuSCs	Cardiotoxin injection	Tibialis anterior	Muscle contractility improvement	[32]
Mice	Human MDSCs	Cryolesion	Tibialis anterior	Fusion with host myofibers	[34]
SD rats	Autologous MSCs	Open crush trauma	Soleus muscle	Muscle force improvement	[44]
SD rats	Autologous BM-MSCs	Open crush trauma	Soleus muscle	Contraction force increase	[45]
SD rats	Autologous BM-MSCs	Open crush trauma	Soleus muscle	Muscle force improvement	[46]
Wistar rats	Autologous ADSCs	Surgical laceration	Soleus muscle	Regenerating myofibers increase	[47]
Wistar rats	Autologous BM-MSCs	Scalpel laceration	Adductor brevis	Regenerating myofibers increase	[48]
Mice	BM-MSCs	Contusion	Gastrocnemius muscle	Muscle fibrosis and inflammation	[49]

SC, stem cell, Mdx mice, dystrophin-deficient mice, MuSCs, muscle satellite stem cells, MDSCs, muscle-derived stem cells, SD, Sprague Dawley rat, MSCs, mesenchymal stem cells, BM, bone marrow, ADSCs, adipose tissue-derived stem cells.

**Table 2:** Macrophages and skeletal muscle regeneration. In vivo studies.

Animals	Injury	Muscle	Depletion Strategy	Results	Ref
Mice	Notexin	Tibialis anterior	Diphtheria toxin	M1-MPs, switching in M2-MPs	[58]
Mice	Cardiotoxin	Tibialis anterior	-	M1- MPs, switching in M2-MPs	[62]
Mice	Laceration	Gastrocnemius	-	M1/M2 phenotype-like classification	[63]
Mice	Cardiotoxin	Gastrocnemius	-	Phenotype transition	[64]
Mouse	Cardiotoxin	Tibialis anterior	-	AMPK⍺1 involved in M2 polarization	[65]
Mice	Cardiotoxin	Tibialis anterior	Diphtheria toxin	SkMR impairment	[66]
Wistar rats	Bupivacaine	Tibialis anterior	Cl_2_MDP liposome & γ-rays	MP number decrease	[67]
Mice	Cooled probe	Tibialis anterior	Clodronate liposomes	Regeneration impairment	[68]
Mice	Cooled probe	Tibialis anterior	-	Muscle strength recovery impairment	[69]
Mice	FAE	Hindlimb muscles	-	Necrotic myofiber persistence	[70]
Mice	Barium Chloride	Quadriceps	-	Necrotic myofiber persistence	[71]
Mice	Barium Chloride	Quadriceps	-	CCL2 for immune cell recruitment	[72]
Mice	FAE	Hindlimb muscles	-	Necrotic myofiber persistence fat accumulation occurrence	[73]
Mice	TK-I/R	Gastrocnemius	-	Muscle functionalities recover by M1-MPs	[74]

MPs, macrophages, MKP-1, mitogen-activated protein kinase phosphatase-1, AMPK⍺1, protein kinase AMP-activated catalytic subunit α-1, Cl_2_MDP, dichloromethylene diphosphonate, FAE, femoral artery excision, CCl2, C-C motif chemokine ligand 2, BaCl_2_, barium chloride, TK-I/R, tourniquet-induced ischemia/reperfusion injury.

**Table 3:** Macrophages and myogenic cells precursors: a functional crosstalk. In vitro and in vivo studies.

	**Cell Culture**			**Results**	**Ref**
In vitro	MPCs/MPs co-culture			MPs rescue MPCs from spontaneous apoptosis	[76]
	MPCs/MPs co-culture			Direct contacts between MPs on MPCs are not required	[78]
	**Graft**	**Muscle**	**Injury**	**Results**	**Ref**
In vivo	Mice	Tibialis anterior	Notexin injection	MPs and MPCs anti-apoptotic contacts establishment	[76]
	Human	Vastus lateralis	Electrically stimulation	Different spatial position of MPs in regenerating areas	[78]
	Wistar rats	Tibialis anterior	Surgery ablation	MPs conditioned medium enhances SkMR	[79]

MPCs, myogenic precursors cells, MPs, macrophages, SkMR, Skeletal muscle regeneration

## Data Availability

Not Applicable.
